# Late failure of endovascular repair of an isolated true aneurysm of the profunda femoris artery

**DOI:** 10.1016/j.jvscit.2026.102127

**Published:** 2026-01-05

**Authors:** Kathryn Pillai, Andrew Son, Sheela Patel, Ahmed M. Abou-Zamzam

**Affiliations:** a School of Medicine, California University of Science and Medicine, Colton, CA; b Division of Vascular Surgery, Kaiser Permanente Riverside Medical Center, Riverside, CA; c University of California, Irvine Medical Center, Orange, CA; d Division of Vascular Surgery, Loma Linda University Health, Loma Linda, CA

**Keywords:** Profunda femoris artery aneurysm (PFAA), Endovascular repair, Partial aneurysmectomy

## Abstract

Isolated profunda femoris artery aneurysms (PFAAs) are rare among all peripheral artery aneurysms. True PFAAs are often associated with atherosclerosis, whereas PFA pseudoaneurysms are related to trauma. The optimal treatment for PFAAs is unclear, but a variety of methods have been used, such as open resection with or without revascularization, stent graft placement, and coil embolization. We present an interesting case of a PFAA initially treated with endovascular means with a late failure requiring open surgery nearly 3 years after the initial treatment.

Isolated profunda femoris artery aneurysms (PFAAs) are rare among all peripheral artery aneurysms. While accounting for only 1.0% to 2.6%^1^ of femoral artery aneurysms, PFAAs are associated with a 30% to 45% risk of rupture.^2^ The rarity of true PFAA is thought to be owing to the anatomy of the PFA deeply embedded in the thigh muscles, which leads to less shear stress. An estimated 45% to 81%^3^ of cases of true PFAAs are found in conjunction with other arterial aneurysms such as aortic, iliac, and superficial femoral.^4^ The PFAAs are often asymptomatic and discovered as an incidental finding on imaging. When symptomatic, PFAAs may exhibit compressive symptoms such as neurapraxia, swelling, and pain. True PFAAs are often associated with atherosclerosis, whereas PFA pseudoaneurysms are often related to trauma.^5^ Optimal treatment of PFAAs is unclear, but a variety of methods have been used, such as ligation or resection with or without revascularization, resection with revascularization, stent graft placement, and coil embolization.^6^ We present an interesting case of a PFAA initially treated with endovascular means with late failure requiring open surgical treatment.

## Case report

**Fig 1:**
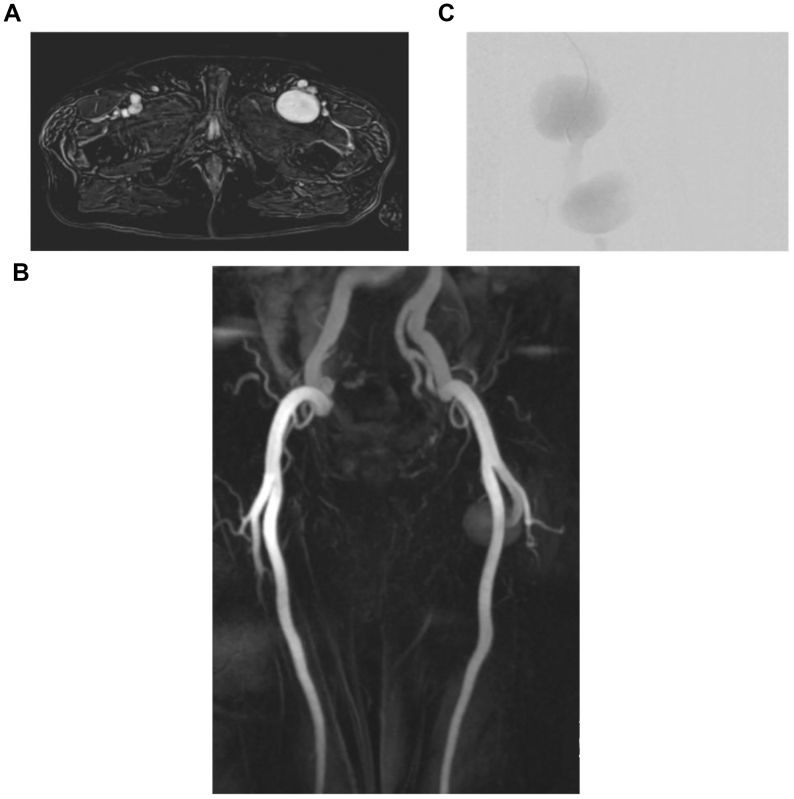
**(A)** Initial magnetic resonance angiography coronal view of the lower extremity that shows a left profunda femoris aneurysm. **(B)** Initial magnetic resonance angiography axial view of the pelvis that shows a left profunda femoris aneurysm. **(C)** Conventional angiogram that shows a 9.1-cm bilobed configuration of the left profunda femoris aneurysm.

A 53-year-old male patient presented to the emergency department owing to acute kidney injury on chronic kidney disease with a 6.1 potassium value found during an outpatient clinic visit. His past medical history was significant for coronary artery disease and an angiomyolipoma of the left kidney. The patient had undergone a left radical nephrectomy 5 years before his presentation. A computed tomography (CT) scan of the abdomen/pelvis without contrast incidentally found a bilobed left profunda femoral artery aneurysm. This was confirmed by Doppler ultrasound examination. Further imaging with magnetic resonance angiography of the pelvis and left thigh showed a large 9.1-cm bilobed left femoral aneurysm originating from the proximal left deep femoral artery (Fig 1, *A* and *B*). The aneurysm was initially thought to be a pseudoaneurysm because the patient had a history of kidney artery embolization for nephrectomy via a femoral approach (although the side of access was uncertain). An infectious etiology was excluded through a white blood cell scan.

**Fig 2:**
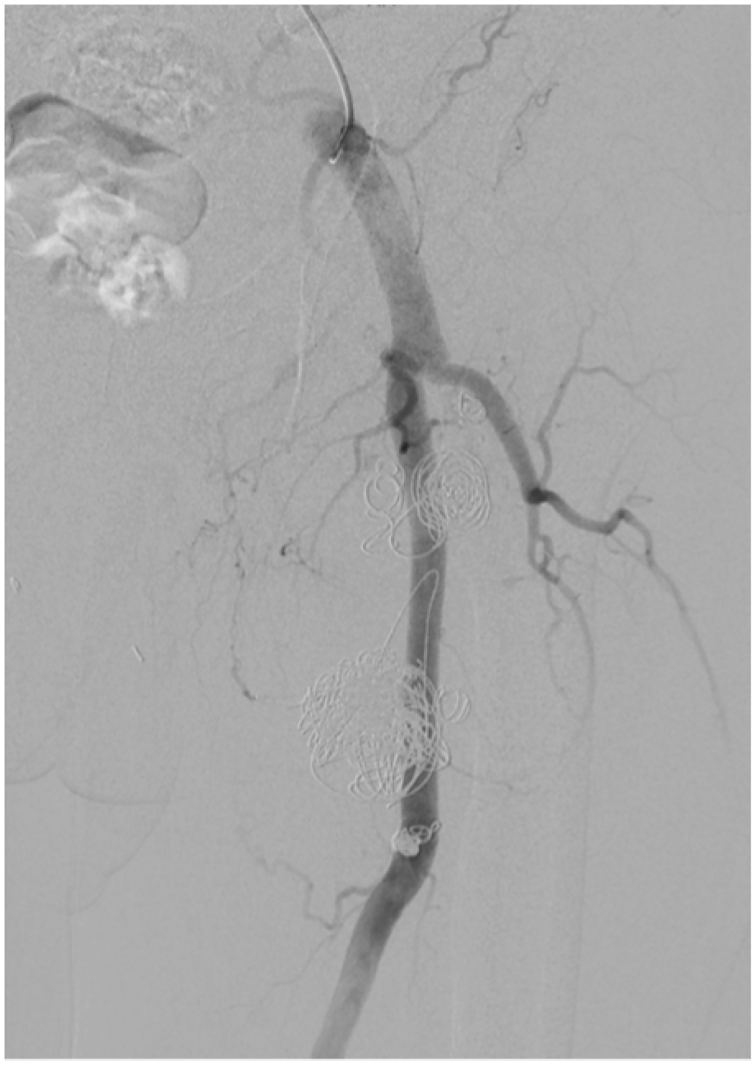
Completion angiogram that shows left profunda femoris aneurysm exclusion with coils.

Owing to the large size of the aneurysm, the patient's comorbidities and the lack of mass effect, endovascular treatment was planned. The patient was taken for diagnostic angiography. Imaging demonstrated a bilobed PFAA with a short intervening normal segment of artery. The superior aspect of the aneurysm originated from the main profunda branch. The inferior aspect of the aneurysm was larger in size. Extensive mural thrombus was present, and a side branch outflow artery was seen in the inferior lobe (Fig 1, *C*). A single side-branch outflow from the lower lobe of the aneurysm was selectively coiled before filling the lower lobe with larger coils. Subsequently, the superior aspect of the aneurysm was filled with embolization coils, and an Amplatzer II plug was deployed in the main profunda inflow branch. In total, the PFAA was treated with a mixture of 37 Cook Medical Nester, Cook Medical Tornado, and Terumo Azur coils. A completion angiogram (Fig 2) demonstrated no flow into the inferior aneurysm, and low flow into the superior aneurysm, although the patient was heparinized at the time. There were no intraoperative complications. After coil embolization, the proximal PFA trunk and a major early branch remained patent with exclusion of flow into the bilobed aneurysm. There may have been a wisp of flow around the Amplatzer plug, but the patient was fully heparinized at this point. The patient recovered quickly and was discharged on aspirin and apixaban (Eliquis) owing to an acute right upper extremity deep vein thrombosis. The patient remained on apixaban for 3 months.

**Fig 3:**
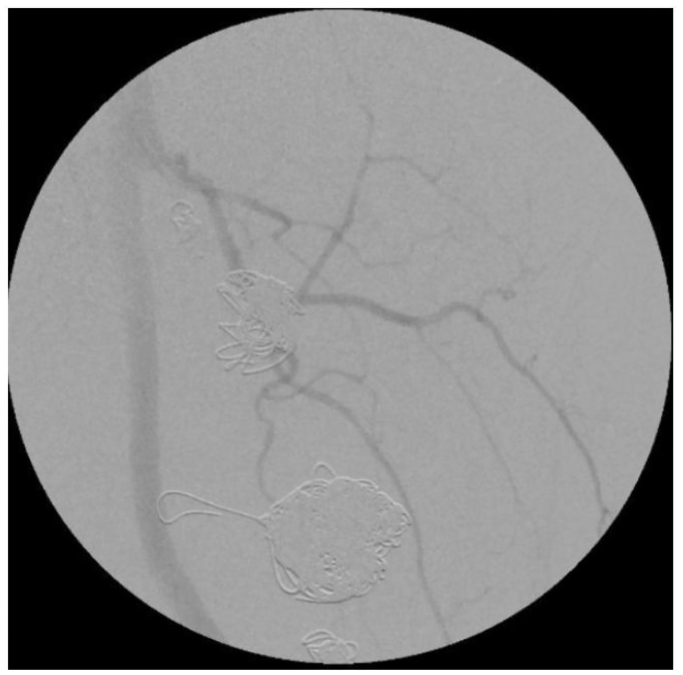
Angiogram that shows left profunda femoris aneurysm with apparent continued exclusion with coils.

Doppler ultrasound examination of the left lower extremity 5 months later demonstrated no significant size increase, but there was some patchy arterial flow noted. Owing to continued flow, the patient was offered another intervention. There was a delay of several months when the patient presented 7 months after the original procedure. At that time, an angiogram demonstrated thrombosis of both lobes of the PFAA without any filling of contrast into the sac on delayed imaging. The angiogram also demonstrated the coils and Amplatzer plug (Fig 3). Color Doppler ultrasound examination confirmed no flow into the aneurysms; therefore, no additional intervention was undertaken.

**Fig 4:**
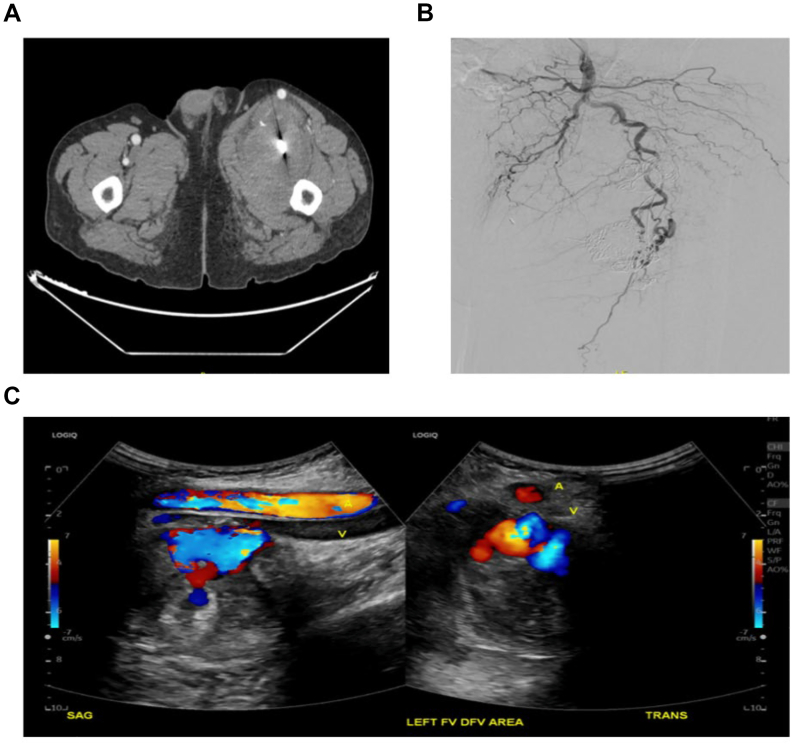
**(A)** Computed tomography angiography (CTA) of left thigh with left profunda femoris aneurysm with significant size increase but no filling. **(B)** Conventional angiogram that showed no flow into the sac of the aneurysm. **(C)** Duplex ultrasound examination showing internal residual flow in the inferior portion of the bilobed aneurysm.

The patient did well but was lost to follow-up. Two and one-half years later and 32 months status post endovascular treatment of the PFAA, the patient presented to our clinic with complaints of left thigh swelling and pain, with shooting pain radiating down his thigh just past his knee which was worse with walking. CT angiography (CTA) showed no flow into aneurysms (Fig 4, *A*). A left lower extremity angiogram showed no opacification in the aneurysms (Fig 4, *B*). There was a presence of a pulsatile femoral mass. Venous duplex ultrasound examination demonstrated no evidence of deep venous thrombosis. An arterial duplex ultrasound examination showed redemonstration of the bilobed aneurysm, with the larger, inferior aneurysm having residual arterial flow (Fig 4, *C*).

Owing to the clinical picture of a mass effect and the concern for residual flow seen on Doppler ultrasound examination, the patient was taken to the operating room. Operative exposure was obtained via a standard vertical femoral incision that was extended distally to allow complete anterior exposure of the aneurysm. With the superficial femoral artery retracted medially, the main profunda trunk was controlled, as were a few proximal outflow profunda branches. Anterior exposure of the palpable bilobed aneurysm was gained. After inflow control of the proximal profunda trunk, the aneurysm was opened without distal control. There was extensive hematoma, and the prior coils were encountered. After evacuation of the hematoma and coils, there was noted to be brisk arterial bleeding from two feeding branches—one into each of the lobes of the aneurysm sac. These feeding branches were oversewn with 5-0 and 4-0 Prolene. After evacuation and ligation, there was no evidence of bleeding into the aneurysm sac, and it was confirmed to be a true PFAA with a bilobed configuration. There was no evidence of in-line flow into the PFAA from the profunda trunk and the proximal Amplatzer plug was left in situ. The pathology report and cultures demonstrated no evidence of connective tissue disorder nor chronic infection from the aneurysm wall and sac.

Two weeks after the open repair, the patient experienced a wound dehiscence that required operative debridement with delayed primary closure. He subsequently recovered well with steady improvement in his left leg weakness and resolution of the left foot drop. An arterial duplex ultrasound examination of the left lower extremity 2 months after open repair demonstrated no evidence of residual aneurysm with patent left lower extremity arteries from the external iliac to the pedal arteries. The patient was seen in clinic 6 months later ambulating with no assist devices with resolution of his left lower extremity edema and weakness, complete wound healing, no femoral mass, and a palpable pedal pulse. The patient consented to the publication of this report.

## Discussion

Because true PFAAs are rare, there is no established protocol for indications for surgical repair. A case series reported a recommendation of ≥20 mm in diameter or symptomatic PFAA for surgical repair.^7^ Typically, when PFAAs are found, they are symptomatic owing to their location, and surgical repair is warranted owing to the high likelihood of rupture. Here, our patient's bilobed aneurysm was initially discovered incidentally on a CT scan of the abdomen/pelvis. Owing to its large size at discovery, endovascular intervention via coiling and plug placement was done with initial good results. Endovascular intervention was elected over open surgery owing to the location of the aneurysm distal to the main trunk of the profunda femoris, the presence of a patent superficial femoral artery, and the patient's multiple comorbidities. The anticipated low morbidity and quick recovery from an endovascular procedure were also preferred by the patient. Additionally, there was a hope that endovascular intervention would protect against damage that might occur to collateral vessels during open surgery.^1^

The late return of the patient with symptomatic progress 2.5 years later stresses the importance of routing monitoring after endovascular interventions. The patient's clinical status with compressive symptoms of swelling, pain, and neurapraxia made the decision to intervene very straightforward. The reliability of cross-sectional imaging, such as CTA, was disappointing in this case. CT is often the first line in imaging for evaluation of aneurysms and its use is recommended for surgical planning. However, in this patient, CTA failed to identify a leak. Indeed, Doppler ultrasound examination demonstrated the pertinent findings of residual flow into the aneurysm. Because CTA is widely used in the diagnosis of arterial aneurysms, with ultrasound examination and magnetic resonance angiography as second- and third-line imaging, the limitations of imaging must be recognized.^6^ Duplex ultrasound examination can be an inexpensive and valuable adjunct to cross-sectional imaging; this is especially true in cases where prior embolization coils may limit visualization.

Most reported repairs of true PFAAs describe resection and revascularization with bypass, although ligation or resection without revascularization is also described.^6^ Endovascular repair is tempting owing to its less invasive nature, but, when failure occurs, open repair is often definitive. Endovascular therapies on the PFA also must be used with great caution in the setting of SFA occlusive disease.^2^ Although endoleaks are widely described in aortic repair, there is little in the literature demonstrating the deleterious outcomes after endovascular repair of a PFAA. Endoleaks and endotensions should be carefully monitored as illustrated in this case with a type II endoleak presenting with significant symptoms >2.5 years after apparently successful treatment.

## Conclusions

True aneurysms of the PFA are rare. Treatment with endovascular means (embolization, plugs, and stenting) may be attempted. Close monitoring for late complications such as continued growth and compression symptoms is paramount. Duplex ultrasound examination is useful in follow-up when gold standard imaging results are equivocal. Endoleaks and endotensions should be considered as explanations for late failure and open surgical treatment can be safely performed with durable results.

## Funding

None.

## Disclosures

None.
